# Effectiveness of Hydrodilatation for Adhesive Capsulitis in Patients With Pre-existing Rotator Cuff Tears

**DOI:** 10.7759/cureus.98492

**Published:** 2025-12-04

**Authors:** Aishwarya Prakash, Hussain Selmi, Shiva Namdeo, Tressa Amirthanayagam, Daoud Makki

**Affiliations:** 1 Trauma and Orthopaedics, Watford General Hospital, West Hertfordshire Teaching Hospitals NHS Trust, Watford, GBR

**Keywords:** adhesive capsulitis, diabetic frozen shoulder, frozen shoulder, hydrodilatation, rotator cuff tears

## Abstract

Background

Adhesive capsulitis (AC) is a chronic, debilitating condition characterized by pain and progressive stiffness of the shoulder joint. Hydrodilatation (HD) is a commonly used method of non-surgical management. However, its effectiveness in patients with rotator cuff pathology remains unclear.

Objective

The study aimed to evaluate and compare the clinical effectiveness of HD in patients with AC, with and without rotator cuff tears. A secondary objective was to assess clinical outcomes in patients with concurrent type 2 diabetes mellitus.

Methods

This retrospective cohort comprised 78 patients (six excluded due to loss to follow-up and two excluded due to incomplete data) who underwent HD between 2021 and 2024. Patients were stratified by rotator cuff integrity (intact n=60, partial-thickness tear n=10, full-thickness tear n=8). Pain (Visual Analog Scale (VAS)) and range of motion (ROM: abduction, flexion, and external rotation) were recorded at baseline, six weeks, three months, and six months. Change scores were analyzed using the Wilcoxon signed-rank and Kruskal-Wallis tests with Benjamini-Hochberg false discovery rate (FDR) correction. The primary endpoint was the change in abduction at three months.

Results

All groups demonstrated significant within-group improvements in pain and ROM (all p-values adjusted for multiple comparisons using the Benjamini-Hochberg FDR (p(FDR < 0.05), except flexion at six months in the full-thickness tear group (p(FDR) = 0.271). At three months, median abduction improvement was 80° (60-90) in the intact group vs 40° (20-75) in partial-thickness and 35° (17.5-70) in full-thickness tears (H=13.21, p(FDR)=0.005). External rotation gains were also greater in the intact group, most notably at three months (Z=3.91, p(FDR) = 0.002 vs partial-thickness). Pain reduction was observed in all groups, but reductions were greater in the intact versus full-thickness subgroup (three months: Z=−3.08, p(FDR)=0.014). Patients with diabetes demonstrated attenuated ROM recovery but similar pain improvement compared with non-diabetic patients.

Conclusions

HD provided meaningful pain relief across all patient subgroups in this retrospective cohort. However, rotator cuff integrity substantially influenced ROM recovery, with reduced gains in patients with partial- and full-thickness tears. Diabetic patients appeared to achieve less ROM improvement, although this subgroup was small, and findings should be interpreted cautiously. Pre-procedure imaging and individualised counselling are recommended.

## Introduction

Adhesive capsulitis (AC) affects ~2%-5% of the population and presents with progressive, painful restriction of the glenohumeral joint [1,2]. Diagnosis is typically clinical after excluding other shoulder pathology. Hydrodilatation (HD) is frequently employed when initial conservative measures (e.g., physiotherapy) are insufficient, though outcomes vary, likely reflecting procedural technique, rehabilitation, and patient factors [3-7]. Surgical options such as manipulation under anaesthesia (MUA) and arthroscopic capsular release are generally reserved for refractory disease.

HD is an image-guided capsular distension procedure. Benefit is thought to derive from hydraulic capsular expansion, with the necessity of capsular rupture remaining debated [8,9]. Recurrence patterns and outcomes appear to vary by aetiology in prior work [10]. Evidence specifically addressing HD effectiveness in the presence of rotator cuff tears is limited. In older adults, cuff pathology may reduce distension efficacy by permitting fluid extravasation and reducing intra-articular pressure [11]. In routine clinical practice, many patients are treated for a clinical diagnosis of AC without undergoing pre-procedure imaging, and rotator cuff tears are often only identified incidentally during image-guided intervention.

Objective and hypothesis

We evaluated HD outcomes in AC patients with incidental rotator cuff tears compared with those without tears. Our a priori hypothesis was that full-thickness tears would be associated with attenuated range of motion (ROM) gains relative to partial-tear and no-tear cohorts. Consistent with contemporary trials, we focused on early- to mid-term outcomes (six weeks to six months), where clinically relevant change is typically greatest [12,13].

## Materials and methods

Design and setting

This was a retrospective cohort study of consecutive patients undergoing image-guided HD at West Hertfordshire Teaching Hospitals NHS Trust in Watford, England, between January 2021 and December 2024 (48-month recruitment period).

Eligibility

Patients were identified through electronic medical record (EMR) review using procedural codes for image-guided HD. Clinical eligibility was verified retrospectively through documented assessment by extended scope practitioners (specialist shoulder physiotherapists). Inclusion required a clinical diagnosis of AC, defined by documented progressive painful restriction of passive glenohumeral ROM in multiple planes with negative radiographs, and treatment with HD after failure of conservative management (minimum six weeks of physiotherapy and oral analgesia).

Exclusion criteria were previous ipsilateral shoulder surgery, glenohumeral arthritis, inflammatory arthropathy, rotator cuff tears requiring primary surgical repair (based on documented tear size, retraction, patient age, and functional demands), and prior HD of the affected shoulder.

Rotator cuff classification

Pre-procedure ultrasound categorised shoulders as intact cuff (no tear), partial-thickness tear (< 100% tendon thickness), or full-thickness tear (complete tendon discontinuity with communication to the subacromial space).

HD protocol and rehabilitation

All procedures were performed by musculoskeletal radiologists using a standardised institutional protocol under fluoroscopic guidance. With the patient supine, the glenohumeral joint was accessed via the rotator interval. Intra-articular positioning was confirmed with 5 mL iodinated contrast (visible under fluoroscopy) via a 21-gauge needle. A mixture of 40 mg of triamcinolone acetonide and 10 mL of 0.25% levobupivacaine was injected, followed by 20 mL of 0.9% saline using an "inject-and-relax" technique to distend the capsule. Patients rested the arm for 48 hours (avoiding axial loading and overhead activity) and commenced a standardised physiotherapy program consisting of weekly 30-minute sessions for eight weeks, then as clinically indicated.

Outcomes and follow-up

Outcomes were abstracted from clinic records at baseline, six weeks, three months, and six months. Pain was measured with the Visual Analog Scale (VAS; 0-10 scale). ROM included flexion, abduction, and external rotation.

All ROM assessments were performed by extended scope practitioners using a standardised passive goniometric protocol that remained unchanged throughout the study period. The protocol specified patient positioning (supine for all measurements), scapular stabilization technique (examiner's hand firmly stabilizing the scapula to isolate glenohumeral motion), goniometer placement landmarks (center of rotation at the glenohumeral joint, stationary arm aligned with the thorax for flexion/abduction or parallel to the spine for ER, moving arm aligned with the humerus), and passive end-range determination (gentle pressure to capsular endpoint without pain provocation). Practitioners underwent annual competency assessment to ensure technique consistency. All measurements were recorded to the nearest 5°.

Follow-up appointments occurred at six weeks, three months, and six months post procedure.

Rationale for the six-month follow-up

Peak clinical benefit from HD and intra-articular corticosteroid is typically observed between six and 12 weeks and remains informative for approximately six months [12]. Contemporary trials frequently use three- to six-month primary endpoints [14,15], and systematic reviews define "mid-term" outcomes as 12 weeks to 12 months. Accordingly, we prespecified a three-month primary endpoint with secondary outcomes extending to six months.

Statistical analysis

The primary endpoint was delta (Δ) abduction at three months, when HD with corticosteroid demonstrates peak efficacy. Secondary endpoints were Δ flexion, Δ ER, and Δ VAS at six weeks, three months, and six months to assess early response and treatment durability. Change scores were defined as Δ = follow-up value − baseline value. Shapiro-Wilk tests indicated non-normality across all outcomes and timepoints (all p < 0.05); therefore, a non-parametric analysis plan was adopted.

Within-group comparisons were performed using the Wilcoxon signed-rank test. Between-group differences were evaluated using the Kruskal-Wallis test. Where omnibus tests were significant, post-hoc pairwise comparisons were conducted using Wilcoxon rank-sum tests (intact cuff vs partial-thickness tear, intact cuff vs full-thickness tear, and partial-thickness vs full-thickness tear). The Benjamini-Hochberg false discovery rate (FDR) correction was applied separately to (i) within-group tests, (ii) between-group omnibus tests, and (iii) pairwise comparisons. A two-sided α of 0.05 was used throughout. Analyses were performed using R (R Foundation for Statistical Computing, Vienna, Austria) with the tidyverse package (Posit PBC, Boston, MA, USA) and the rstatix package (developed by Alboukadel Kassambara).

Ethics and governance

This project was conducted as a service evaluation under institutional governance in accordance with UK Health Research Authority guidelines. Formal Research Ethics Committee review was not required for retrospective analysis of routinely collected de-identified clinical data. All patients provided informed consent for clinical care and data use.

## Results

Cohort

Eighty-six patients underwent HD during the study period, of whom six were excluded due to loss to follow-up and two due to incomplete data, leaving 78 patients in the analytic cohort. The mean symptom duration before HD was approximately six months. Mean age at intervention was 55 years (range 36-81 years). Rotator cuff status was asymmetrically distributed, reflecting the natural prevalence of incidental cuff pathology in our population with AC: intact cuff n=60 (77%), partial-thickness tear n=10 (13%), and full-thickness tear n=8 (10%). Type 2 diabetes mellitus was present in nine patients (11.5% of the cohort).

Baseline passive ROM differed across tear types. In the intact cuff group, mean flexion was 53.8°, abduction 36.4°, and external rotation 17.9°. In partial-thickness tears, mean flexion was 42.0°, abduction 41.5°, and external rotation 18.0°. In full-thickness tears, mean flexion was 60.0°, abduction 35.0°, and external rotation 13.8°.

**Figure 1:**
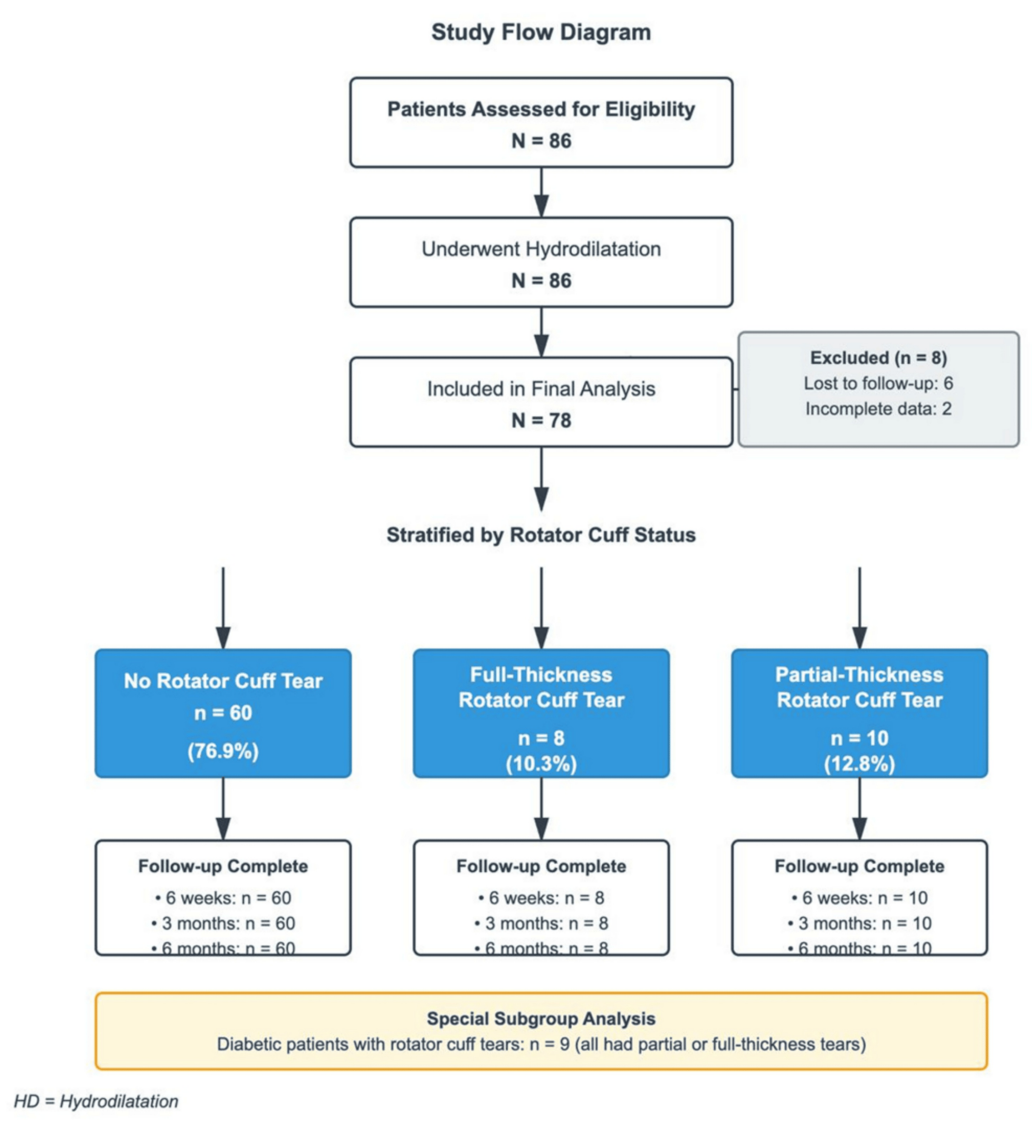
Study flow diagram HD: hydrodilatation

Figure 1 illustrates the study flow and subgroup allocation.

Capsular rupture

Capsular rupture during HD was not systematically documented in radiology reports during the study period, and we were therefore unable to quantify its frequency across rotator cuff subgroups. As a result, the relationship between capsular rupture and clinical response in our cohort remains speculative.

Primary endpoint: change in abduction at three months

Median improvements in abduction at three months favoured the no-tear subgroup: 80° (60-90) (no tear) vs 40° (20-75) (partial-thickness) vs 35° (17.5-70) (full-thickness). Omnibus and pairwise tests were significant after FDR adjustment, as detailed below. The between-group difference at 3 months was statistically significant (H = 13.21, p(FDR) = 0.005).

Within-group changes (FDR-adjusted)

All subgroups demonstrated significant improvements in ROM and VAS at six weeks, three months, and six months (all p-values adjusted for multiple comparisons using the Benjamini-Hochberg FDR correction (p(FDR) < 0.05), except flexion at six months in the full-thickness subgroup (p(FDR) = 0.271). In the no-tear subgroup, abduction and external rotation improvements remained highly statistically significant at all timepoints (all p(FDR) < 0.001). Partial-tear patients also demonstrated significant gains, with p(FDR) values ranging from 0.010 to 0.013.

Within-group results are summarised in Table 1, which shows Wilcoxon signed-rank test statistics and FDR-adjusted p-values for each subgroup and time point.

**Table 1:** Within-group change (Wilcoxon signed-rank test) ABD: abduction; ER: external rotation; FLEX: flexion; VAS: Visual Analog Scale pain score; p(FDR): p-value adjusted for multiple comparisons using the Benjamini-Hochberg False Discovery Rate correction; 6W: six weeks; 3M: three months; 6M: six months Very small p-values are reported as p < 0.001.

Group	Outcome	Comparison	N pairs	Z statistic	p (FDR)
No tear	ABD	Pre vs 6W	60	-6.74	5.82e-11
		Pre vs 3M	60	-6.74	5.82e-11
		Pre vs 6M	60	-6.74	5.82e-11
	ER	Pre vs 6W	60	-6.74	5.82e-11
		Pre vs 3M	60	-6.74	5.82e-11
		Pre vs 6M	60	-6.74	5.82e-11
	FLEX	Pre vs 6W	60	-6.74	6.89e-11
		Pre vs 3M	60	-6.74	6.72e-11
		Pre vs 6M	60	-3.66	0.001
	VAS pain	Pre vs 6W	60	-6.74	5.82e-11
		Pre vs 3M	60	-6.74	5.82e-11
		Pre vs 6M	60	-6.74	5.82e-11
Partial tear	ABD	Pre vs 6W	10	-2.80	0.011
		Pre vs 3M	10	-2.80	0.011
		Pre vs 6M	10	-2.80	0.011
	ER	Pre vs 6W	10	-2.67	0.012
		Pre vs 3M	10	-2.52	0.013
		Pre vs 6M	10	-2.52	0.013
	FLEX	Pre vs 6W	10	-2.80	0.011
		Pre vs 3M	10	-2.80	0.011
		Pre vs 6M	10	-2.52	0.013
	VAS pain	Pre vs 6W	10	-2.80	0.011
		Pre vs 3M	10	-2.80	0.011
		Pre vs 6M	10	-2.80	0.011
Full tear	ABD	Pre vs 6W	8	-2.52	0.023
		Pre vs 3M	8	-2.52	0.023
		Pre vs 6M	8	-2.20	0.036
	ER	Pre vs 6W	8	-2.52	0.017
		Pre vs 3M	8	-2.52	0.017
		Pre vs 6M	8	-2.52	0.017
	FLEX	Pre vs 6W	8	-2.52	0.023
		Pre vs 3M	8	-2.52	0.023
		Pre vs 6M	8	-1.09	0.271
	VAS pain	Pre vs 6W	8	-2.52	0.017
		Pre vs 3M	8	-2.52	0.017
		Pre vs 6M	8	-2.52	0.017

Table 2 compares the change in abduction, external rotation, flexion, and pain scores across the three rotator cuff groups (no-tear, partial-thickness tear, and full-thickness tear). Statistically significant between-group differences were observed for abduction at all timepoints (six weeks, three months, and six months), with the no-tear group demonstrating larger improvements than both tear groups. External rotation also differed significantly between groups at each assessment point, again favouring the no-tear group. Flexion showed significant group differences at six weeks and at three months, but these differences were no longer evident by six months, suggesting partial convergence of functional gains over time.

**Table 2:** Between-Group Differences in Change Scores (Kruskal–Wallis Test) All groups compared: no tear, partial tear, full tear ABD: abduction; ER: external rotation; FLEX: flexion; p(FDR): p-value adjusted for multiple comparisons using the Benjamini–Hochberg False Discovery Rate correction; 6W: six weeks; 3M: three months; 6M: six months Very small p-values are reported as p < 0.001.

Outcome	Timepoint	H statistic	p (FDR)
ABD	6W	15.06	0.003
	3M	13.21	0.005
	6M	12.42	0.006
ER	6W	6.94	0.034
	3M	18.01	0.001
	6M	8.01	0.022
FLEX	6W	8.09	0.022
	3M	11.99	0.006
	6M	4.97	0.083
VAS pain	6W	8.59	0.020
	3M	11.21	0.007
	6M	10.67	0.008

Post-hoc pairwise comparisons for significant omnibus findings are presented in Table 3.

**Table 3:** Pairwise differences in delta (Δ; Wilcoxon rank-sum test) ABD: abduction; ER: external rotation; FLEX: flexion; VAS: Visual Analog Scale pain score; p(FDR): p-value adjusted for multiple comparisons using the Benjamini-Hochberg False Discovery Rate correction. Very small p-values are reported as p < 0.001.

Outcome	Timepoint	Pair	Z statistic	p (FDR)
ABD	6W	No tear vs Partial	2.99	0.017
	3M	No tear vs Partial	2.93	0.018
	6M	No tear vs Partial	2.64	0.028
ER	6W	No tear vs Partial	2.40	0.037
	3M	No tear vs Partial	3.91	0.002
	6M	No tear vs Partial	2.37	0.037
FLEX	6W	No tear vs Full	2.67	0.028
	3M	No tear vs Full	3.26	0.014
VAS pain	6W	No tear vs Full	-2.84	0.018
	3M	No tear vs Full	-3.08	0.014
	6M	No tear vs Full	-2.92	0.017

Diabetes subgroup

In an exploratory subgroup analysis, patients with diabetes tended to demonstrate numerically smaller ROM gains, while pain improvement was comparable to non-diabetic patients. Mean improvements in ROM in diabetic and non-diabetic groups are presented in Table 4; no formal statistical comparison was performed because of the small subgroup size. This pattern is consistent with the recognised diabetic shoulder phenotype characterised by capsular fibrosis and reduced tissue compliance. 

**Table 4:** Diabetes subgroup (diabetic n = 9; non-diabetic n = 69) ABD: abduction; ER: external rotation; FLEX: flexion; ROM: range of motion; 6W: six weeks; 3M: three months; 6M: six months

Diabetic status	Timepoint	ABD (°)	ER (°)	FLEX (°)
Diabetic	6W	27	14	19
	3M	25	18	19
	6M	22	18	18
Non-diabetic	6W	72	29	57
	3M	76	37	61
	6M	74	34	54

Clinical magnitude (Δ medians IQR)

**Figure 2:**
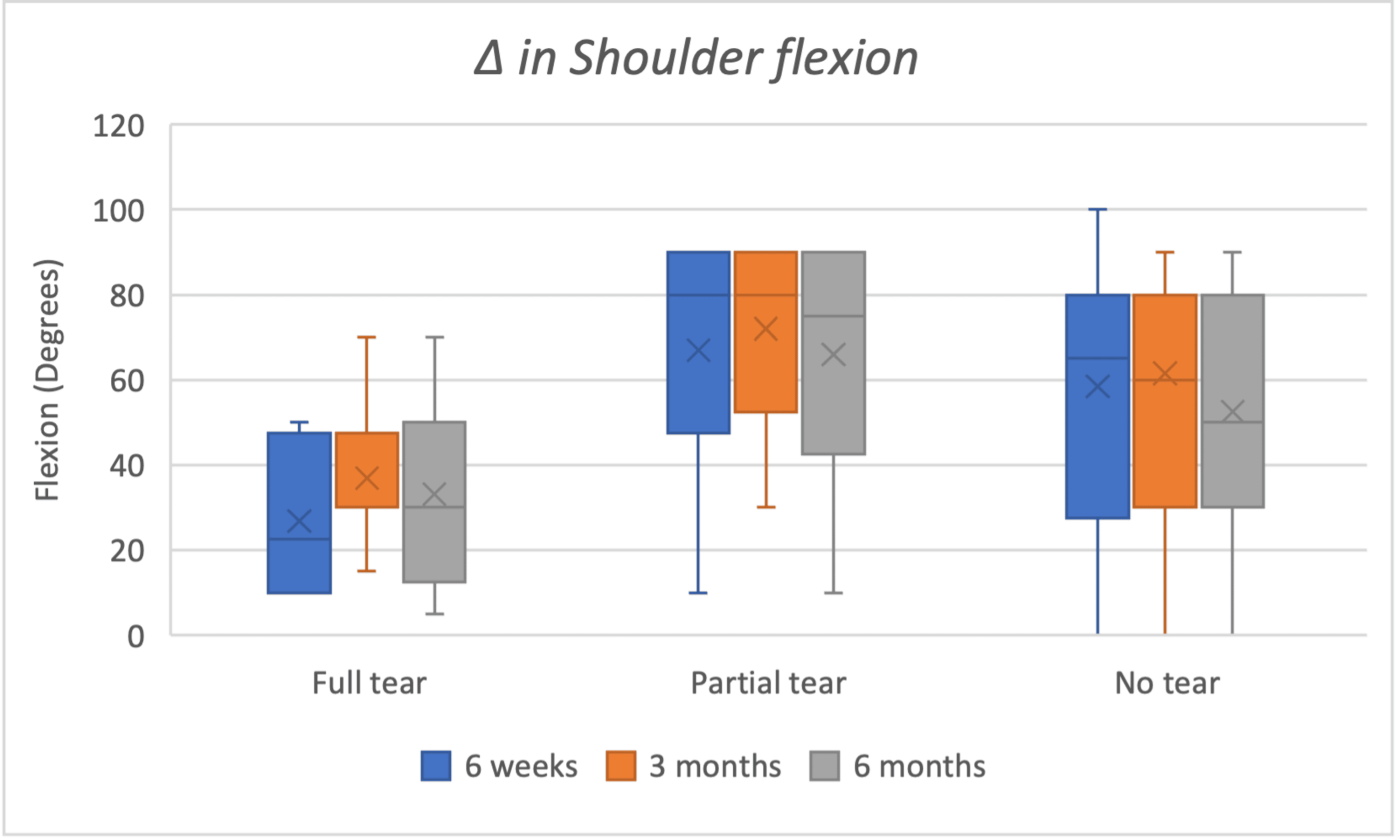
Box whisker chart showing improvements in shoulder flexion at six weeks, three months and six months Δ: delta; Full-thickness tear n=8; Partial thickness tear n=10; No tear n=60

**Figure 3:**
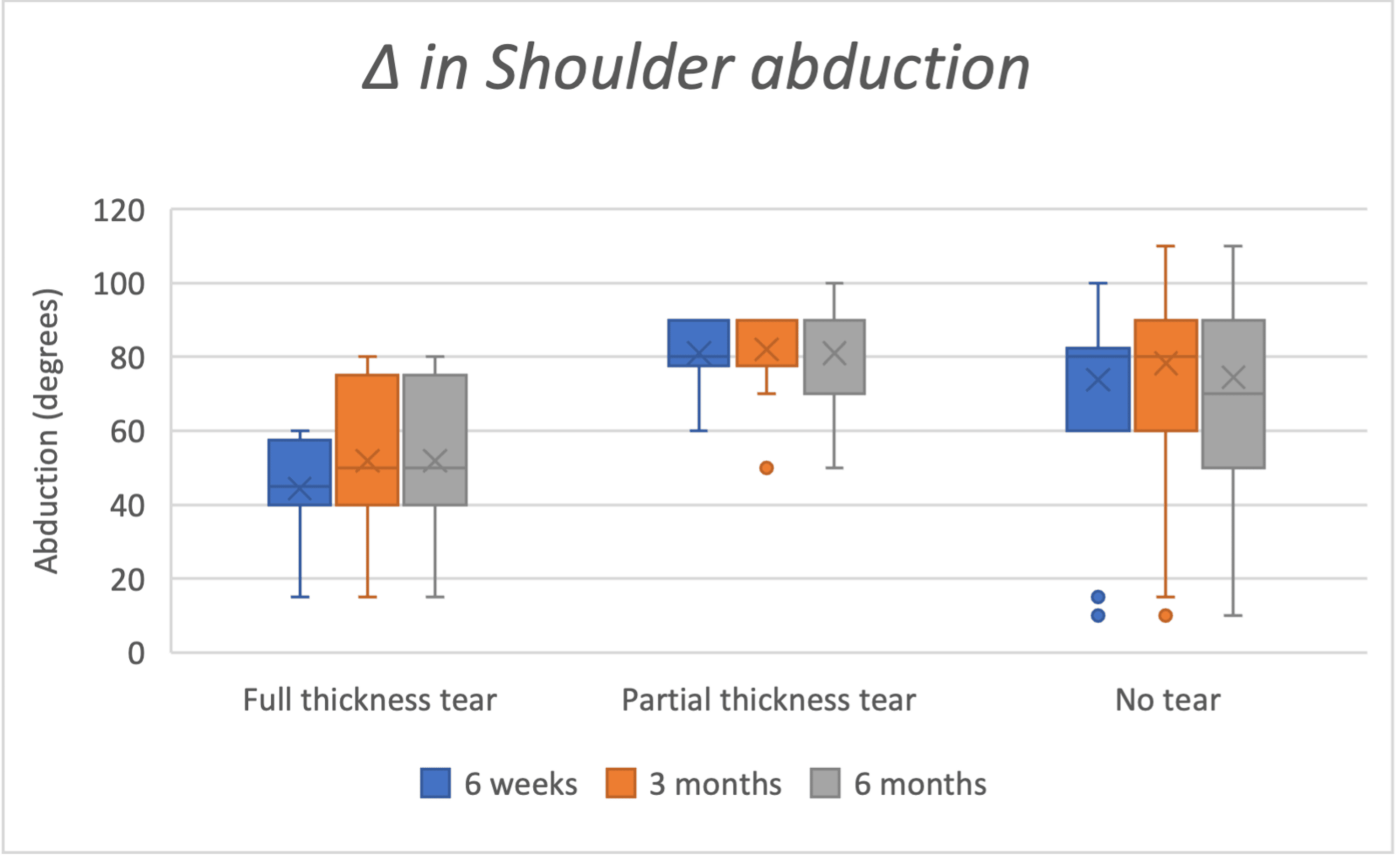
Box whisker chart showing improvements in shoulder abduction at six weeks, three months, and six months Δ: delta; Full-thickness tear n=8; Partial thickness tear n=10; No tear n=60

**Figure 4:**
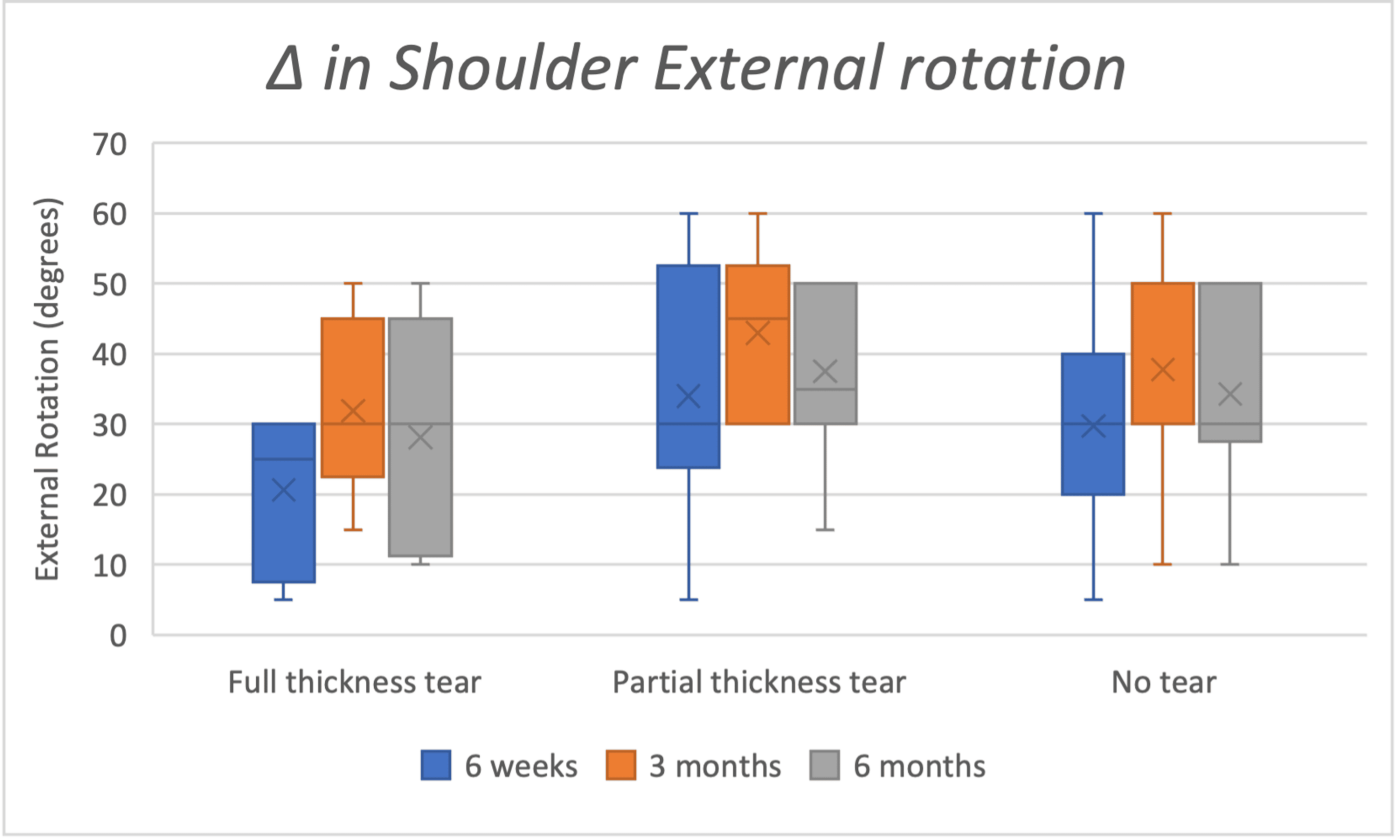
Box whisker chart showing improvements in shoulder external rotation at six weeks, three months, and six months Δ: delta; Full-thickness tear n=8; Partial thickness tear n=10; No tear n=60

At three months, Δ abduction medians were 80° (60-90) in no-tear, 40° (20-75) in partial-thickness, and 35° (17.5-70) in full-thickness groups. Δ VAS medians were approximately six (no-tear), five (partial-thickness), and four (full-thickness). Box-and-whisker plots of improvements in ROM in flexion, abduction, and external rotation are shown in Figures 2-4.

**Figure 5:**
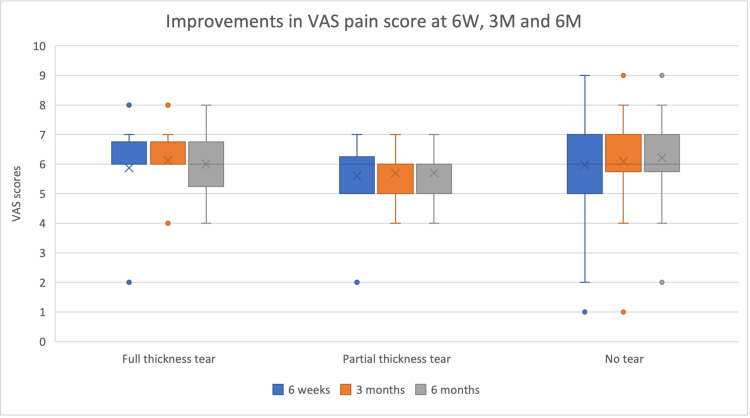
Improvements in Visual Analog Scale pain score six weeks (6W), three months (3M), and six months (6M) Full-thickness tear n=8; Partial thickness tear n=10; No tear n=60

Pain scores differed significantly across groups at all timepoints, with the no-tear group demonstrating greater pain reduction, particularly compared with patients with full-thickness tears (Figure 5). These findings support a consistent pattern in which rotator cuff integrity influences the degree of functional recovery following HD, while analgesic benefit remains present across all subgroups.

## Discussion

In this retrospective cohort of patients with AC treated with HD, pain and ROM improved across all subgroups, with smaller ROM gains in those with rotator cuff tears, particularly full-thickness, while analgesic benefit was preserved. These findings align with the recognised mechanisms of HD: mechanical benefit from capsular distension depends on increased intra-articular pressure, which cuff defects may compromise through preferential extravasation of injectate into the subacromial bursa [11]. Simultaneously, corticosteroid-mediated suppression of inflammatory mediators is likely responsible for consistent pain reduction across groups [12, 13]. Functional improvement peaking at approximately three months with attenuation by six months is consistent with the well-described temporal response to intra-articular corticosteroid therapy [14, 15].

Previous HD literature has largely focused on procedural technique rather than concomitant pathology. For example, capsule-preserving versus capsule-rupturing distension approaches show similar analgesic benefit but potentially different capsular stretch mechanics [4]. Meta-analyses and randomised trials demonstrate HD superiority over physiotherapy alone and comparable outcomes to manipulation under anaesthesia in selected patients [6, 13, 16]. However, many studies explicitly excluded rotator cuff tears [4, 15], leaving uncertainty about generalizability to common real-world presentations. Makki et al. found comparable outcomes across AC etiologies but did not stratify by tear integrity [17]. Park et al. reported diminished HD benefit in elderly patients with tears, though without a quantitative comparison across tear grades [11]. The present study extends this literature by directly comparing early- to mid-term outcomes across no-tear, partial-thickness, and full-thickness subgroups with statistical adjustment for multiple comparisons.

The present findings suggest that rotator cuff integrity modulates the mechanical efficacy of HD, even when tears are incidental, and patients are selected for nonoperative management. This supports the biomechanical hypothesis that loss of capsular pressurisation limits capsular stretch despite appropriate injection volumes [11]. In terms of capsular integrity during HD itself, our data did not allow us to robustly distinguish outcomes according to capsular rupture status. However, it is plausible that in shoulders with intact cuffs, higher intra-articular pressures and more uniform capsular stretch can be achieved before either capsular yield or fluid extravasation occurs. Conversely, in the presence of cuff defects, earlier decompression of the injectate into the subacromial space may limit effective capsular distension even if capsular rupture is achieved. These mechanistic considerations remain speculative in our cohort and would require dedicated imaging or pressure-monitoring studies to confirm. Importantly, analgesic benefit was preserved across all groups, implying that corticosteroid-driven biochemical effects may be less dependent on structural integrity [12,13]. The differential ROM outcomes across rotator cuff subgroups despite identical corticosteroid dosing suggest that mechanical factors related to capsular integrity modulate treatment response. The intermediate outcomes in the partial-tear group, despite preserved capsular integrity, suggest that factors beyond simple fluid extravasation may influence HD effectiveness, including altered tendon compliance, concurrent cuff pathology, or pain-mediated limitation of early rehabilitation.

Regarding diabetes, systematic reviews confirm that individuals with diabetes have approximately fivefold higher prevalence of AC [18, 19] and may experience poorer outcomes [19, 20]. Our results are consistent with this literature: attenuated ROM gains with preserved pain relief [12, 13]. We did not perform formal statistical comparisons between diabetic and non-diabetic subgroups because of the small diabetic sample (n=9); the observed differences in ROM should therefore be regarded as exploratory. In our cohort, diabetic patients showed smaller mean gains in flexion, abduction, and external rotation than non-diabetic patients at each time point (Table 4), despite similar pain reduction. This is biologically plausible given the known diabetic shoulder phenotype characterised by cytokine overexpression, collagen cross-linking, and fibroblastic dysregulation [21-24]. In our cohort, diabetes often coexisted with rotator cuff pathology, suggesting a compounded risk profile where metabolic and structural factors may converge.

Clinically, these findings endorse pre-procedure imaging, particularly in older or diabetic populations, to guide expectation-setting and follow-up planning. HD remains appropriate in these groups, but ROM improvement may be less pronounced and slower to recover.

Limitations

This study has several limitations that inform the interpretation of the findings. The retrospective design limits control over confounders such as symptom duration, prior treatments, and comorbid shoulder pathology; therefore, observed associations cannot be interpreted as causal. Subgroup sizes, particularly for full-thickness (n=8) and partial-thickness tears (n=10), reduce statistical power and increase susceptibility to Type II error and result in fragility, with the asymmetrical distribution (intact n=60 versus partial n=10 versus full n=8) increasing the risk of confounding by unmeasured baseline differences. Follow-up was limited to six months, reflecting the interval at which corticosteroid and HD effects are most clinically pronounced [14, 15] but preventing evaluation of long-term durability, recurrence risk, or onward surgery rates.

Outcome measures were restricted to ROM and VAS pain scores. These are clinically meaningful endpoints, but do not capture health-related quality of life or shoulder-specific functional performance; validated patient-reported outcome measures such as the Oxford Shoulder Score would have provided a more comprehensive appraisal. Although rotator cuff status was ultrasound-based, HD was performed under fluoroscopic guidance, and procedural variation (e.g., final injected volume achieved) was not quantified, which may influence capsular distension. We did not systematically record procedural parameters such as maximum injectate volume, intra-articular pressure, or capsular rupture status, which may influence capsular distension and treatment response. There is no ultrasound anatomical differentiation of rotator cuff pathology. Other systemic comorbidities, such as thyroid disease and dyslipidemia, were not systematically recorded and therefore could not be analysed. In patients with diabetes, we did not collect HbA1c or other measures of glycemic control, so we were unable to assess whether diabetic control modified the response to HD. Missing data from patients lost to follow-up were handled using available-case analysis without imputation; per-timepoint sample sizes are provided in tables.

Our study design does not permit separation of the corticosteroid effect from the mechanical distension effect, as all patients received both components as part of standard HD. Isolation of their individual contributions would require a randomised trial comparing HD with corticosteroid versus intra-articular corticosteroid injection alone. Despite these constraints, the study reflects real-world clinical pathways and incorporates rigorous statistical control for multiple testing to minimise false-positive findings.

Implications and future directions

Pre-procedure imaging and patient-specific counselling are advisable when cuff pathology or diabetes is present. Future prospective studies should recruit larger tear subgroups, incorporate standardised patient-reported outcome measures, consider technique modifications (e.g., injection site/volume), and extend follow-up to evaluate recurrence and durability. We specifically recommend randomised controlled trials comparing HD with corticosteroid versus corticosteroid injection alone to definitively establish the mechanical contribution of capsular distension.

## Conclusions

In this retrospective cohort, HD was associated with significant pain reduction in AC across rotator cuff subgroups and in patients with and without diabetes. ROM gains were modulated by cuff integrity and appeared attenuated in full-thickness tears and in patients with diabetes. Given the small numbers in some subgroups, particularly the diabetic cohort, these observations should be considered hypothesis-generating. Pre-procedure imaging and individualized counselling are recommended to optimize selection, expectations, and rehabilitation planning.

## References

[1] Neviaser TJ (1987). Adhesive capsulitis. Orthop Clin North Am.

[2] Sarasua SM, Floyd S, Bridges WC, Pill SG (2021). The epidemiology and etiology of adhesive capsulitis in the U.S. Medicare population. BMC Musculoskelet Disord.

[3] Anton HA (1993). Frozen shoulder. Can Fam Physician.

[4] Pimenta M, Vassalou EE, Klontzas ME, Dimitri-Pinheiro S, Ramos I, Karantanas AH (2024). Ultrasound-guided hydrodilatation for adhesive capsulitis: capsule-preserving versus capsule-rupturing technique. Skeletal Radiol.

[5] Piotte F, Gravel D, Moffet H (2004). Effects of repeated distension arthrographies combined with a home exercise program among adults with idiopathic adhesive capsulitis of the shoulder. Am J Phys Med Rehabil.

[6] Poku D, Hassan R, Migliorini F, Maffulli N (2023). Efficacy of hydrodilatation in frozen shoulder: a systematic review and meta-analysis. Br Med Bull.

[7] Saltychev M, Laimi K, Virolainen P, Fredericson M (2018). Effectiveness of hydrodilatation in adhesive capsulitis of shoulder: a systematic review and meta-analysis. Scand J Surg.

[8] Cho JH (2021). Updates on the treatment of adhesive capsulitis with hydraulic distension. Yeungnam Univ J Med.

[9] Rizk TE, Gavant ML, Pinals RS (1994). Treatment of adhesive capsulitis (frozen shoulder) with arthrographic capsular distension and rupture. Arch Phys Med Rehabil.

[10] Flintoft-Burt M, Stanier P, Planner A, Thahal H, Woods D (2023). Recurrence of the frozen shoulder after hydrodilatation, what is the true incidence?. Shoulder Elbow.

[11] Park YS, Park YH (2015). Adhesive capsulitis in the elderly: comparison of magnetic resonance imaging findings with effectiveness of hydrodilatation treatment. J Korean Geriatr Soc.

[12] Buchbinder R, Green S, Forbes A, Hall S, Lawler G (2004). Arthrographic joint distension with saline and steroid improves function and reduces pain in patients with painful stiff shoulder: results of a randomised, double blind, placebo controlled trial. Ann Rheum Dis.

[13] Gebellí-Jové JT, Buñuel-Viñau A, Canela-Capdevila M, Camps J, Sabench F, Iftimie-Iftimie P (2025). A prospective, randomized, blinded study on the efficacy of using corticosteroids in hydrodilatation as a treatment for adhesive capsulitis of the shoulder. Shoulder Elbow.

[14] Challoumas D, Biddle M, McLean M, Millar NL (2020). Comparison of treatments for frozen shoulder: a systematic review and meta-analysis. JAMA Netw Open.

[15] Paruthikunnan SM, Shastry PN, Kadavigere R, Pandey V, Karegowda LH (2020). Intra-articular steroid for adhesive capsulitis: does hydrodilatation give any additional benefit? A randomized control trial. Skeletal Radiol.

[16] Lädermann A, Piotton S, Abrassart S, Mazzolari A, Ibrahim M, Stirling P (2021). Hydrodilatation with corticosteroids is the most effective conservative management for frozen shoulder. Knee Surg Sports Traumatol Arthrosc.

[17] Makki D, Al-Yaseen M, Almari F, Monga P, Funk L, Basu S, Walton M (2021). Shoulder hydrodilatation for primary, post-traumatic and post-operative adhesive capsulitis. Shoulder Elbow.

[18] Zreik NH, Malik RA, Charalambous CP (2016). Adhesive capsulitis of the shoulder and diabetes: a meta-analysis of prevalence. Muscles Ligaments Tendons J.

[19] Dyer BP, Burton C, Rathod-Mistry T, Blagojevic-Bucknall M, van der Windt DA (2021). Diabetes as a prognostic factor in frozen shoulder: a systematic review. Arch Rehabil Res Clin Transl.

[20] Onggo JD, Gupta M, Low E, Tan LT, Lee KT, Ho SW, Jegathesan T (2025). Hydrodilatation: a comparison between diabetics and non-diabetics with adhesive capsulitis. Int Orthop.

[21] Bunker TD, Reilly J, Baird KS, Hamblen DL (2000). Expression of growth factors, cytokines and matrix metalloproteinases in frozen shoulder. J Bone Joint Surg Br.

[22] Cher JZ, Akbar M, Kitson S (2018). Alarmins in frozen shoulder: a molecular association between inflammation and pain. Am J Sports Med.

[23] Lho YM, Ha E, Cho CH (2013). Inflammatory cytokines are overexpressed in the subacromial bursa of frozen shoulder. J Shoulder Elbow Surg.

[24] Spite M, Clària J, Serhan CN (2014). Resolvins, specialized proresolving lipid mediators, and their potential roles in metabolic diseases. Cell Metab.

